# Analysis of the forced convection of two-phase Ferro-nanofluid flow in a completely porous microchannel containing rotating cylinders

**DOI:** 10.1038/s41598-021-97152-3

**Published:** 2021-09-08

**Authors:** Hamidreza Aghamiri, Mohammadreza Niknejadi, Davood Toghraie

**Affiliations:** Department of Mechanical Engineering, Khomeinishahr Branch, Islamic Azad University, Khomeinishahr Khomeinishahr, Iran

**Keywords:** Engineering, Mathematics and computing, Nanoscience and technology

## Abstract

In the present work, the forced convection of nanofluid flow in a microchannel containing rotating cylinders is investigated in different geometries. The heat flux applied to the microchannel wall is 10,000 W m^−2^. The effects of Reynolds number, the volume fraction of nanoparticles, and the porosity percentage of the porous medium are investigated on the flow fields, temperature, and heat transfer rate. Reynolds number values vary from Re = 250–1000, non-dimensional rotational velocities 1 and 2, respectively, and volume fraction of nanoparticles 0–2%. The results show that increasing the velocity of rotating cylinders increases the heat transfer; also, increasing the Reynolds number and volume fraction of nanoparticles increases the heat transfer, pressure drop, and C_f,ave_. By comparing the porosity percentages with each other, it is concluded that due to the greater contact of the nanofluid with the porous medium and the creation of higher velocity gradients, the porosity percentage is 45% and the values of are 90% higher than the porosity percentage. Comparing porosity percentages with each other, at porosity percentage 90% is greater than at porosity percentage 45%. On the other hand, increasing the Reynolds number reduces the entropy generation due to heat transfer and increases the entropy generation due to friction. Increasing the volume fraction of nanoparticles increases the entropy generations due to heat transfer and friction.

## Introduction

The growing demand for product downsizing in all industrial sectors has been accompanied by global competition. Much work has been done on single-phase heat transfer in microchannels by Tuckerman and Pease^1^ to cool integrated circuits on a very large scale. Today, research in the field of nanofluids has become very extensive. On the one hand, in connection with increasing the thermal conductivity of fluids and increasing the heat transfer, researchers are pursuing the fabrication of nanofluids with nanoparticles and nanotubes with different nanoparticle sizes. In many industrial applications, porous materials play a very important role in the design and development of processes. Currently, the general model of porous media has a very wide field and it can be divided into different sub-categories depending on the type of application, many of which need to be analyzed. To solve many of the above applications, the physics of heat transfer and flow through porous media must be well understood^2^. Fadaei et al.^3^ investigated the forced convection of ferromagnetic nanofluid in a simple channel with a magnetic field and a porous medium. They found that the combined effects of the magnetic field and the porous medium increased the mixing rate and disturbed the thermal boundary layer, which in turn increased the $Nu_{ave}$. Wang and Li^4^ investigated forced convection in a channel containing a porous medium. They found that the use of a porous medium plays an important role in balancing the heat flux of the wall and the heat generated. In addition, with increasing the Darcy number, the $Nu_{ave}$ becomes independent of the porosity property. Li et al.^5^ conducted a theoretical study on forced convection in a circular tube assuming an asymmetric inlet temperature. They found that the $Nu_{ave}$ was strongly dependent on the Pecklet number, and the Biot number. They concluded that the proposed solution could be used as a criterion for numerical problems. Xu and Gong^6^ investigated the forced convection in a tube, part of which is filled with a porous medium. They found that the porosity gradient had no significant effect on the $C_{f,ave}$ and $Nu_{ave}$ decreased with increasing porosity. Baragh et al.^7^ experimentally investigated the convection of fluid in a channel with different porosities. Their results showed that the presence of a porous layer causes more heat transfer of the channel wall to the fluid, which increases the thermal conductivity of the porous material. Li et al.^8^ studied convection in a circular tube with a saturated porous medium. They showed that the Pecklet number and the Biot number play an important role in increasing or decreasing heat transfer. Jamal-Abad et al.^9^ analyzed heat transfer in a tube filled with a porous medium. Considering the thermal conductivity of the porous medium as a linear function of the radius of the tube and using the Brinkman equation, they found that the $Nu_{ave}$ changes as a linear function of the thermal conductivity of the porous medium. Akyildiz and Siginer^10^ proposed a precise solution for the forced convection of a gas flow in a microtube. Heat transfer and flow parameters such as pressure drop ($\Delta P$) were investigated in their research and accurate solutions based on the above parameters were presented, which show an error of less than 5% compared to laboratory results. Zeng et al.^11^ performed a laboratory and numerical study of heat transfer in a heat sink. They showed that with the deformation of the blades, the mixing effect increases significantly. Xu et al.^12^ numerically investigated the convection in a half-filled tube with a porous medium. Their results showed that the parameters of heat transfer and flow resistance within this type of porous foam are highly dependent on porosity, porosity thickness, and porous pore density. Vijay et al.^13^ analyzed the forced convection in open-cell foams. They found that the structure of simple foams could not express the structure of real foams. Hooman et al.^14^ investigated the effects of thermal scattering on the convection in a tube with a saturated porous medium. They showed that using reverse flow and obtaining a closed shape to temperature distribution could have accuracy and error of up to 5%. Shafii and Keshavarz^15^ experimentally investigated the forced convection of a ferromagnetic fluid in a channel with a magnetic and non-magnetic porous medium. Their results showed that the percentage of increase in convective heat transfer coefficient in a magnetic porous medium is 38.66%, while for a non-magnetic porous medium it is 36.13%. Dehghan et al.^16^ analytically investigated the developing flow in a channel filled with a porous medium. For the first time, they provided a relationship for the entrance length in a channel filled with a porous medium. Chen et al.^17^ simulated the convection of nanofluids in the space between two concentric cylinders. Their results showed that at $\phi =$ 10%, heat transfer increased by 23%. Barnoon and Toghraie^18^ investigated the forced convection in space between concentric cylinders, part of which was the porous medium. They found that the porous medium significantly increased heat transfer. Shen et al.^19^ investigated forced convection in a micro heat sink with different porous foams. They found that the effect of the arrangement and location of the porous foam had a significant effect on the temperature and heat transfer properties. Lu et al.^20^ proposed an analytical solution for forced convection in the space between two parallel plates, part of which was a porous medium. They found that the effect of porosity on heat transfer was directly related to the increase in foam thickness from the channel floor. Also, several studies focusing on fluid flow and heat transfer and the types of microtubes, microchannels, and nanofluids, including properties, behavior, and other parameters were reviewed by many researchers^21–39^.

In many of these references, the authors have tried to increase the heat transfer rate with one of several factors mentioned above. Although much attention has been paid to the use of different nanofluids and their effects on heat transfer and the filling of microchannels from the porous medium, unfortunately very little attention has been paid to the placement of the rotating cylinder. The rotation of the cylinders and increasing their number is an innovation of this article.

## Governing equations and solution method

### Problem statement

**Figure 1:**
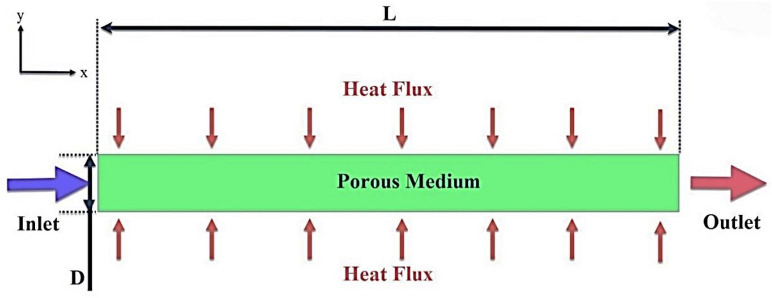
Schematic case A.

**Figure 2:**
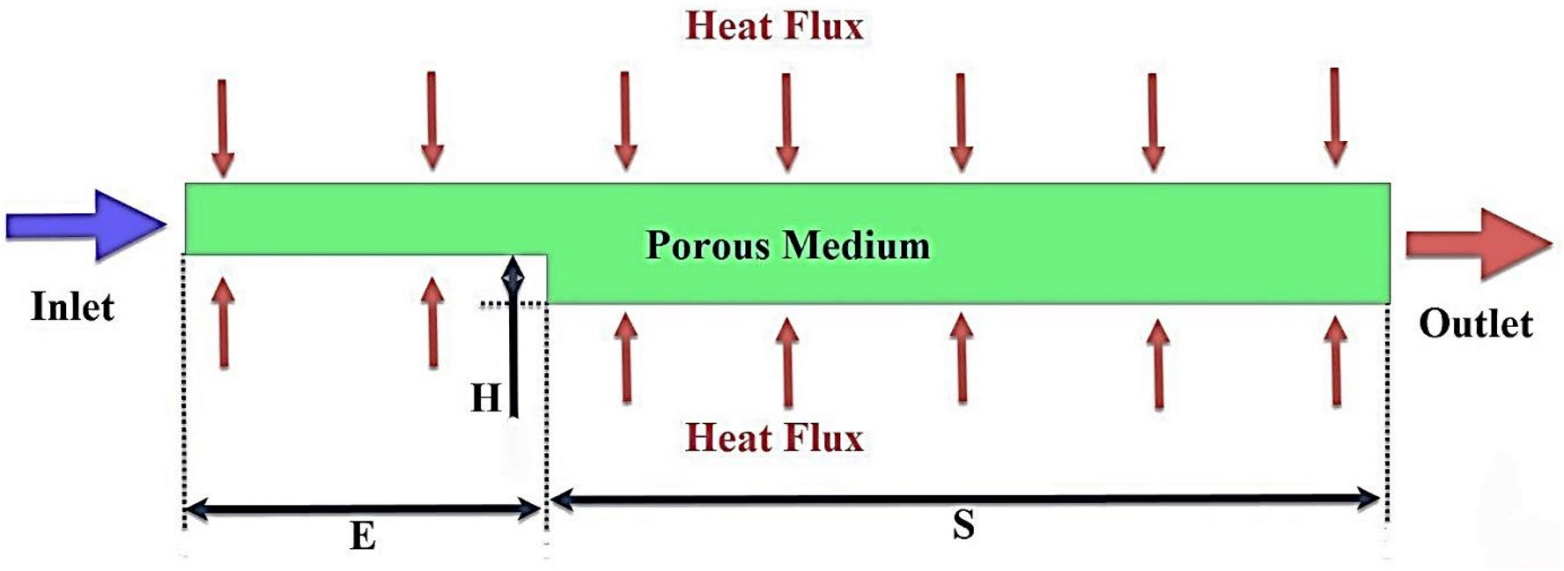
Schematic of case B.

**Figure 3:**
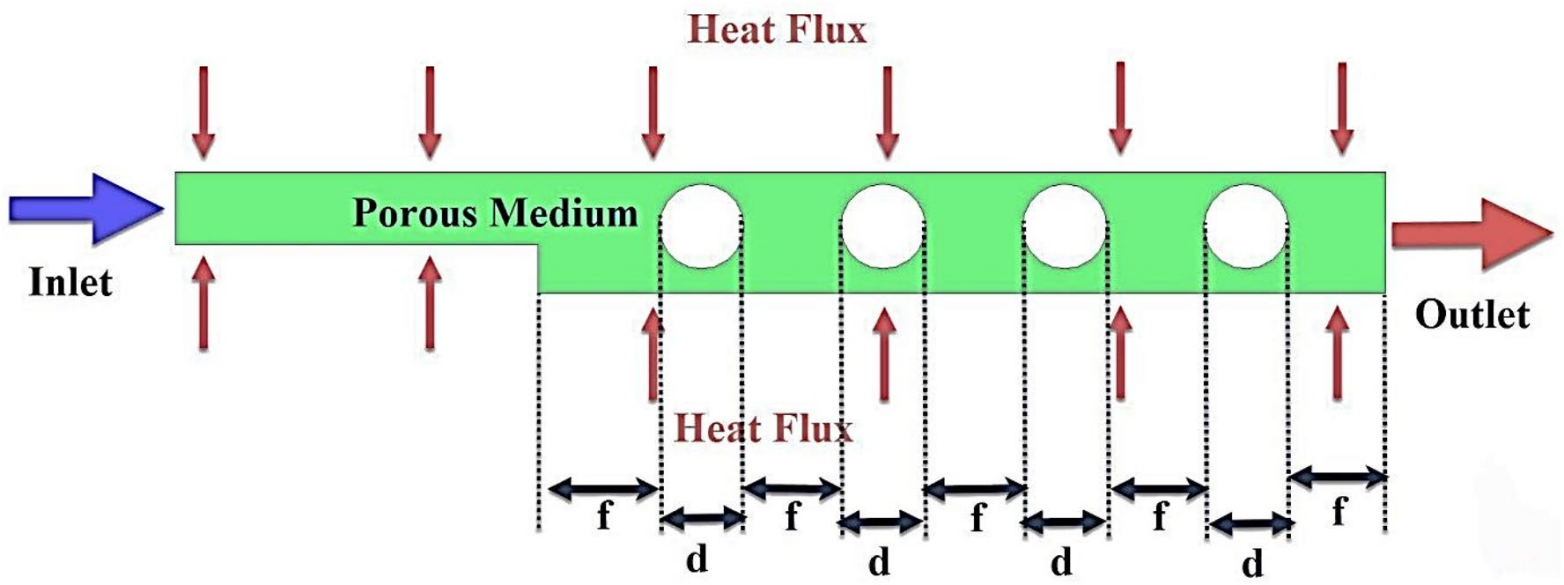
Schematic of case C.

In this study, the effect of $\phi$, Reynolds number, and porosity of porous medium in a microchannel filled with porous medium containing rotating cylinders on the flow field, heat transfer, and entropy generation are investigated. To solve the problem numerically, the CFD method and the finite volume model are used. For higher solution accuracy, the second-order UPWIND method is used and the SIMPLE algorithm is used to couple the pressure and velocity. Also, two-phase mixture model is used. The geometry studied in this research is a two-dimensional microchannel with a diameter of D = 200 $\mu m$ and a length of L = 400 mm, which is filled with a porous medium (see Table 1). The effect of the porous medium placed in the microchannel is analyzed using the Brinkman model at different porosity percentages. In this research, different geometries have been named as a simple microchannel, geometric case A, microchannel with step, geometric case B, and microchannel containing rotating cylinders, geometric case C. The schematic of the problem is shown in Figs. 1, 2 and 3. Figure 2 shows the microchannels with step and Fig. 3 shows the microchannels with step and rotating cylinders. The working fluid at 300 K, Re = 250, 500, 750, and 1000 and $\phi$ = 0, 1, and 2% enters the microchannel filled with a porous medium with $\varepsilon$ = 45 and 90%. The nanoparticles used in this research are iron oxide and the base fluid is also water. The thermophysical properties of which are given in Table 2. A constant heat flux of 10,000 Wm^−2^ is applied to the lower wall of the microchannel.

**Table 1:** Dimensions of geometric cases.

Geometric case	L (mm)	D (μm)	S (mm)	E (mm)	H (μm)	d (μm)	f (mm)
A	400	200	–	–	–	–	–
B	400	200	320	80	20	–	–
C	400	200	320	80	20	150	63.88

**Table 2:** Thermophysical properties of nanoparticles and base fluid^40^.

$\mu \, \, \left[ {kg\,m^{ - 1} \,s^{ - 1} } \right]$	$k\,\left[ {W\,m^{ - 1} \,K^{ - 1} } \right]$	$C_{p} \, \, \left[ {J\,kg^{ - 1} \,K^{ - 1} } \right]$	$\rho \, \, \left[ {kg\,m^{ - 3} } \right]$	
0.001003	0.6	4182	998.2	Water
–	6	670	5200	Iron oxide

### Governing equations

In this research, the Eulerian two-phase mixture model is used to model the water-iron oxide nanofluids. To analyze the porous parts, the Brinkman model, which, unlike the Darcy model, is directly dependent on porosity and viscosity, uses the Local Thermal Equilibrium (LTE) method, in which the temperature of the fluid is equal to the solid surface.

- *Continuty equation *^41^
  1 $$ \overrightarrow {\nabla } .\left( {\rho_{m} \overrightarrow {V}_{m} } \right) = 0, $$
- *Momentum equation *^41^

2 $$ \begin{aligned} \overrightarrow {\nabla } .\left( {\rho_{m} \overrightarrow {V}_{m} \overrightarrow {V}_{m} } \right) & = - \overrightarrow {\nabla } P + \overrightarrow {\nabla } .\left( {\mu_{m} \left( {\overrightarrow {\nabla } \overrightarrow {V}_{m} + \overrightarrow {\nabla } \overrightarrow {V}_{m}^{T} } \right) + \overrightarrow {\nabla } .\left[ {\sum\limits_{k = 1}^{n} {\phi_{k} \rho_{k} \overrightarrow {V}_{dr,k} \overrightarrow {V}_{dr,k} } } \right]} \right) \\ \quad { + }\left( {\frac{{\varepsilon \mu_{m} }}{K} + \frac{{\rho_{m} \varepsilon^{2} C_{f} }}{\sqrt K }\left| {\overrightarrow {V}_{m} } \right|} \right)\overrightarrow {V}_{m} , \\ \end{aligned} $$

where $\rho_{m}$ is the density of the mixture, $\mu_{m}$ is the effective viscosity of the mixture, $\overrightarrow {V}_{m}$ is the velocity vector of the mixture, $\overrightarrow {V}_{dr,k}$ is the velocity vector of the secondary phase, and $C_{f}$ is the shape function. Also,

3 $$ \overrightarrow {V}_{dr,k} = \overrightarrow {V}_{k} - \overrightarrow {V}_{m} , $$

4 $$ C_{F} = 0.55\left( {1 - 5.5\frac{L}{{D_{h} }}} \right). $$

- *Energy equation* ^40^
  5 $$ \overrightarrow {\nabla } \left( {\sum\limits_{k = 1}^{n} {\left( {\rho_{k} C_{p,k} } \right)\phi_{k} \varepsilon \overrightarrow {V}^{k} T} } \right) = \overrightarrow {\nabla } .k_{m} \overrightarrow {\nabla } T, $$

*Drift velocity*:

6 $$ \overrightarrow {V}_{dr,k} = \overrightarrow {V}_{pf} - \sum\limits_{k = 1}^{n} {\frac{{\phi_{k} \rho_{k} }}{{\rho_{m} }}\overrightarrow {V}_{f,k} } , $$

*The mixture velocity* ^40^ *:*

7 $$ \overrightarrow {V}_{m} = \frac{{\sum\limits_{k = 1}^{n} {\phi_{k} \mu_{k} \overrightarrow {V}_{k} } }}{{\rho_{m} }}, $$

*The density of the mixture* ^40^ *:*

8 $$ \rho_{m} = \sum\limits_{k = 1}^{n} {\phi_{k} \rho_{k} } , $$

*The viscosity of the mixture*^40^*:*

9 $$ \mu_{m} = \sum\limits_{k = 1}^{n} {\phi_{k} \mu_{k} } , $$

and

10 $$ \overrightarrow {V}_{pf} = \frac{{\rho_{p} d_{p}^{2} \left( {\rho_{p} - \rho_{m} } \right)}}{{18\mu_{f} f_{drag} \rho_{p} }}\left( {\overrightarrow {g} - \left( {\overrightarrow {V}_{m} .\overrightarrow {\nabla } } \right)\overrightarrow {V}_{m} } \right), $$

11 $$ f_{drag} = \left\{ {\begin{array}{*{20}l} {1 + 0.15{\text{Re}}^{0.687} } \hfill & {{\text{[Re}} \le 1000]} \hfill \\ {0.0183{\text{Re}} } \hfill & {{\text{[Re}} > 1000]} \hfill \\ \end{array} } \right. $$

Therefore, the drift velocity is obtained as a function of relative velocity as follows^40^,

12 $$ \overrightarrow {V}_{dr,k} = \overrightarrow {V}_{pf} - \sum\limits_{k = 1}^{n} {\left[ {\frac{{\phi_{k} \rho_{k} }}{{\rho_{m} }}\overrightarrow {V}_{f,k} } \right]} . $$

### Thermophysical properties of nanofluid^40^

13 $$ \rho_{m} = (1 - \phi )\rho_{f} + \phi \rho_{np} , $$

14 $$ \left( {\rho C_{p} } \right)_{m} = (1 - \phi )\left( {\rho C_{p} } \right)_{f} + \phi \left( {\rho C_{p} } \right)_{np} , $$

15 $$ \mathop \mu \nolimits_{m} = \frac{{\mathop \mu \nolimits_{f} }}{{\mathop {(1 - \phi )}\nolimits^{2.5} }}, $$

16 $$ k_{m} = k_{f} \left[ {1 + 2.72\phi + 4.97\phi^{2} } \right]. $$

### Entropy generation

*Entropy generation due to heat transfer per unit volume*^40^,

17 $$ S_{gen,HT}^{{{\prime \prime \prime }}} = \frac{{k_{m} }}{{T^{2} }}\left[ {\left( {\frac{\partial T}{{\partial x}}} \right)^{2} + \left( {\frac{\partial T}{{\partial y}}} \right)^{2} } \right], $$

*Entropy generation due to friction per unit volume* ^40^ *,*

18 $$ S_{gen,f}^{{{\prime \prime \prime }}} = \frac{{\mu_{m} }}{T}\left[ {2\left( {\left( {\frac{\partial u}{{\partial x}}} \right)^{2} + \left( {\frac{\partial v}{{\partial y}}} \right)^{2} } \right) + \left( {\frac{\partial u}{{\partial y}} + \frac{\partial v}{{\partial x}}} \right)^{2} } \right], $$

*Total entropy generation:*

19 $$ S_{gen,HT} = \int {S_{gen,HT}^{{{\prime \prime \prime }}} } dV, $$

20 $$ S_{gen,f} = \int {S_{gen,f}^{{{\prime \prime \prime }}} dV} , $$

21 $$ S_{gen,Tot} = \int {S_{gen,T}^{{{\prime \prime \prime }}} } dV + \int {S_{gen,F}^{{{\prime \prime \prime }}} } dV. $$

### Measurement parameters

- *Dimensionless parameters*
  22 $$ X = \frac{x}{L}{\text{ , Y = }}\frac{y}{h}{\text{ , U = }}\frac{u}{{u_{in} }}{ , }\Omega = \frac{\omega H}{{2v}}, $$

where x represents the direction of flow and L represents the length of the microchannel.

- *Reynolds number:*
  23 $$ {\text{Re}} = \frac{{\rho_{m} V_{in} L}}{{\mu_{m} }}, $$
- *Pressure drop:*
  24 $$ \Delta P = \frac{{fL\rho_{m} V_{in}^{2} }}{2L}, $$
- *Friction factor:*
  25 $$ C_{f} = \frac{{\tau_{w} }}{{\frac{1}{2}\rho_{m} V_{in}^{2} }}, $$
- *Wall temperature and bulk temperature of nanofluid:*
  26 $$ T_{w} (x) = \frac{1}{A}\int {TdA} , $$
  27 $$ T_{b} (x) = \frac{{\int {T\rho_{m} \left| {Vd\vec{A}} \right|} }}{{\int {\rho_{m} \left| {Vd\vec{A}} \right|} }}, $$
  28 $$ h(x) = \frac{q^{\prime\prime}(x)}{{T_{w} (x) - T_{b} }}, $$
  29 $$ h = \frac{1}{L}\int_{0}^{L} {h(x)dx} , $$
  30 $$ Nu\left( x \right) = \frac{h\left( x \right)L}{{k_{m} }}, $$
  31 $$ Nu_{ave} = \frac{hL}{{k_{m} }}, $$
- *Performance Evaluation Criterion (PEC) *^42^
  31 $$ PEC = \frac{{{\raise0.7ex\hbox{${Nu}$} \!\mathord{\left/ {\vphantom {{Nu} {Nu_{s} }}}\right.\kern-\nulldelimiterspace} \!\lower0.7ex\hbox{${Nu_{s} }$}}}}{{\left( {{\raise0.7ex\hbox{$f$} \!\mathord{\left/ {\vphantom {f {f_{s} }}}\right.\kern-\nulldelimiterspace} \!\lower0.7ex\hbox{${f_{s} }$}}} \right)^{\frac{1}{3}} }}. $$

### Boundary conditions

(A) Inlet conditions:
  The inlet velocity is calculated based on the Reynolds number and the properties of the nanofluid, and the inlet temperature is also constant.
(B) Outlet conditions:
  We do not have temperature conditions and the outlet pressure is equal to the atmospheric pressure.
(C) Conditions of the walls:
  A constant heat flux of 10,000 Wm^−2^ is applied to the upper wall.

### Assumptions

- The flow is steady.
- Viscous dissipation is ignored.
- The base fluid flow is incompressible.
- The base fluid is Newtonian.
- The no-slip condition and no temperature jump are applied in the solid walls.

### Grid independency and validation

**Figure 4:**
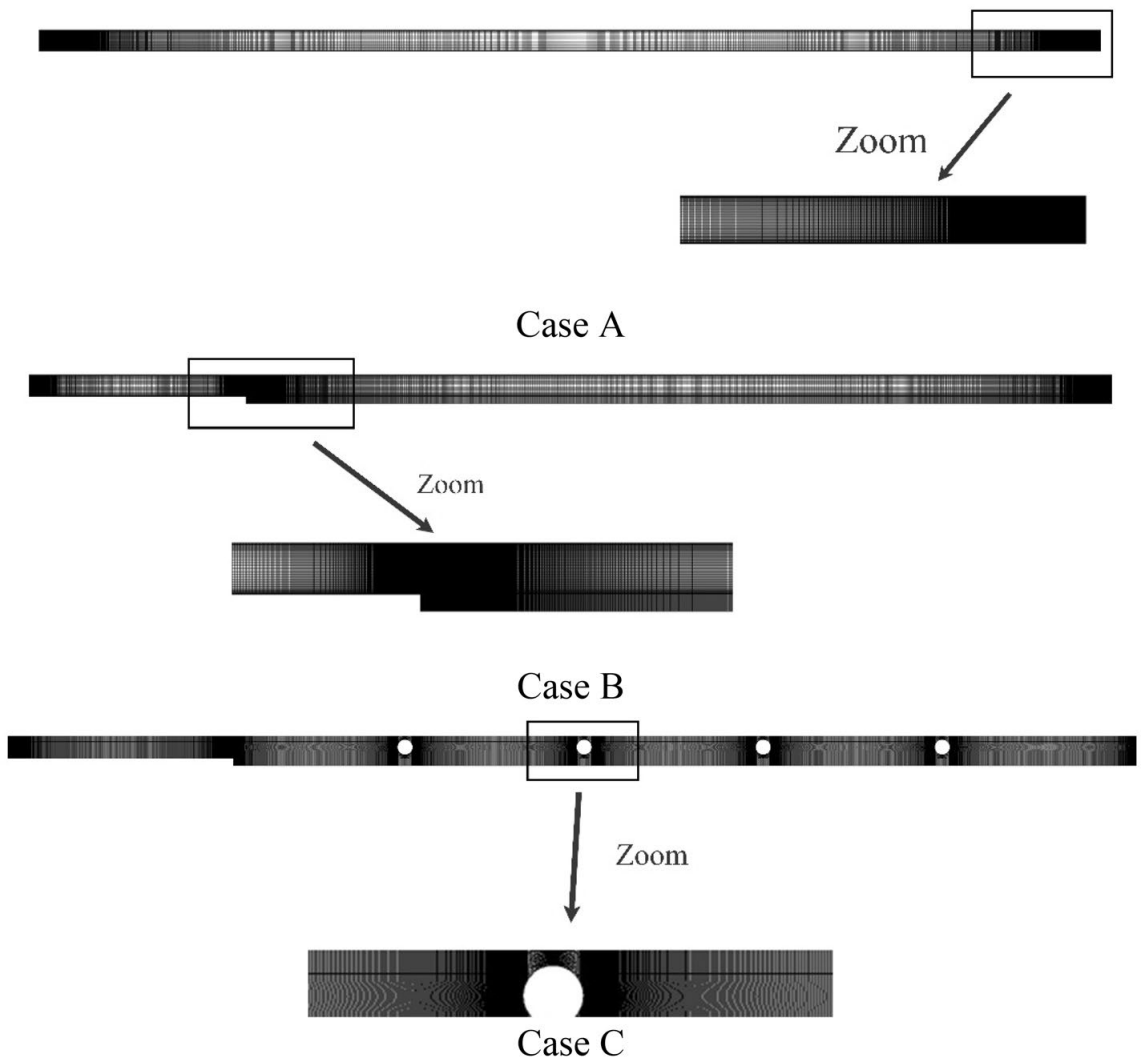
Computational grid.

**Figure 5:**
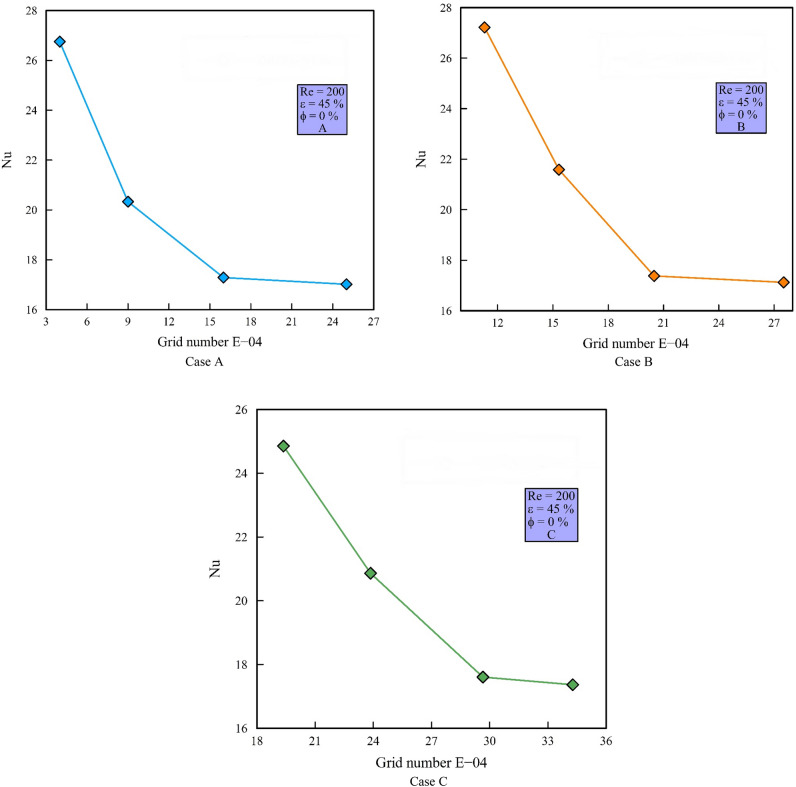
Grid independency diagrams.

**Figure 6:**
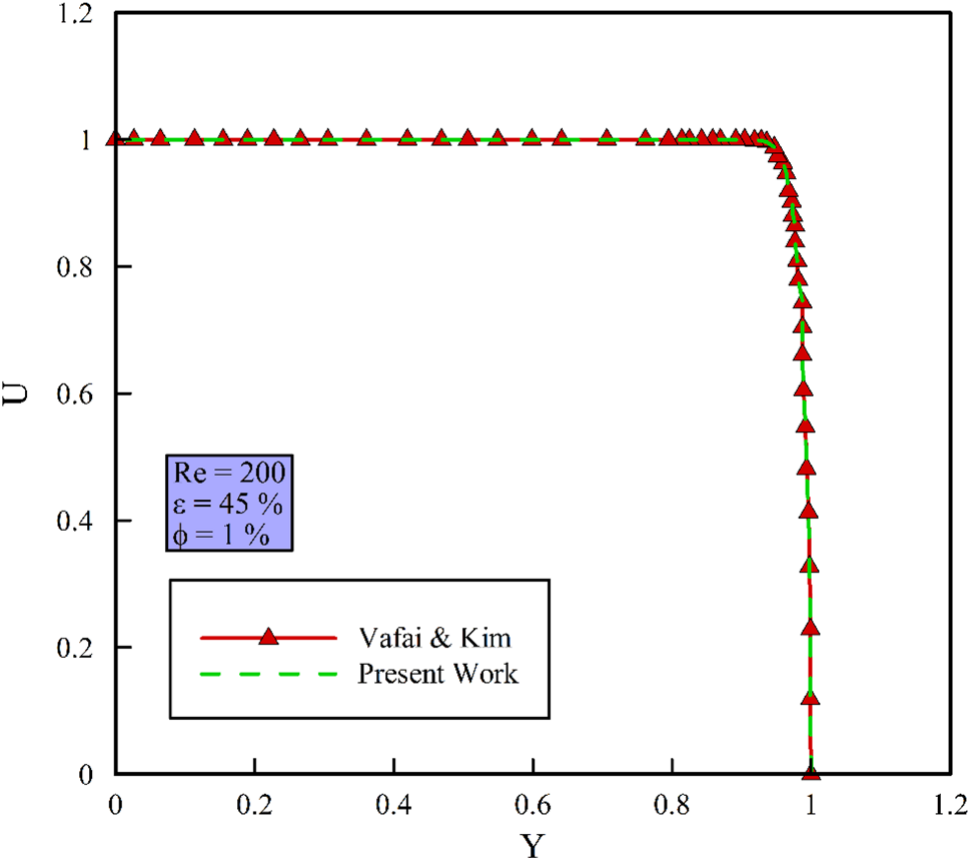
Validation diagram and comparison of the present work with Ref.^43^.

In Fig. 4, a structured grid is used by increasing the number of elements perpendicular to the wall. The independency of the obtained solution from the grid is evaluated at constant heat flux, Re = 200, $\phi$ = 0% and $\varepsilon$ = 45% with $Nu_{ave}$ and continues when the error of each stage falls below 3% compared to the previous stage. Figure 5 shows the $Nu_{ave}$ versus the number of the grids. In the mentioned figures, the difference of $Nu_{ave}$ in points 3 and 4 has reached below 3%; so, point 3 is selected as the desired computational mesh. To ensure the accuracy of the obtained numerical results, it is necessary to compare the obtained numerical results with the valid results of other researchers. For this purpose, Ref.^43^ has been used as the validation reference in Fig. 6. In this reference, half of the velocity profiles at the output are compared with a Re = 200, a porosity of 45%, and $\phi$ = 1%. Because the obtained numerical results are consistent with the experimental results and the error is less than 2%, so the process of numerical problem solving has good accuracy.

## Results and discussion

### Effect the $\phi$ on $\Delta P$

**Figure 7:**
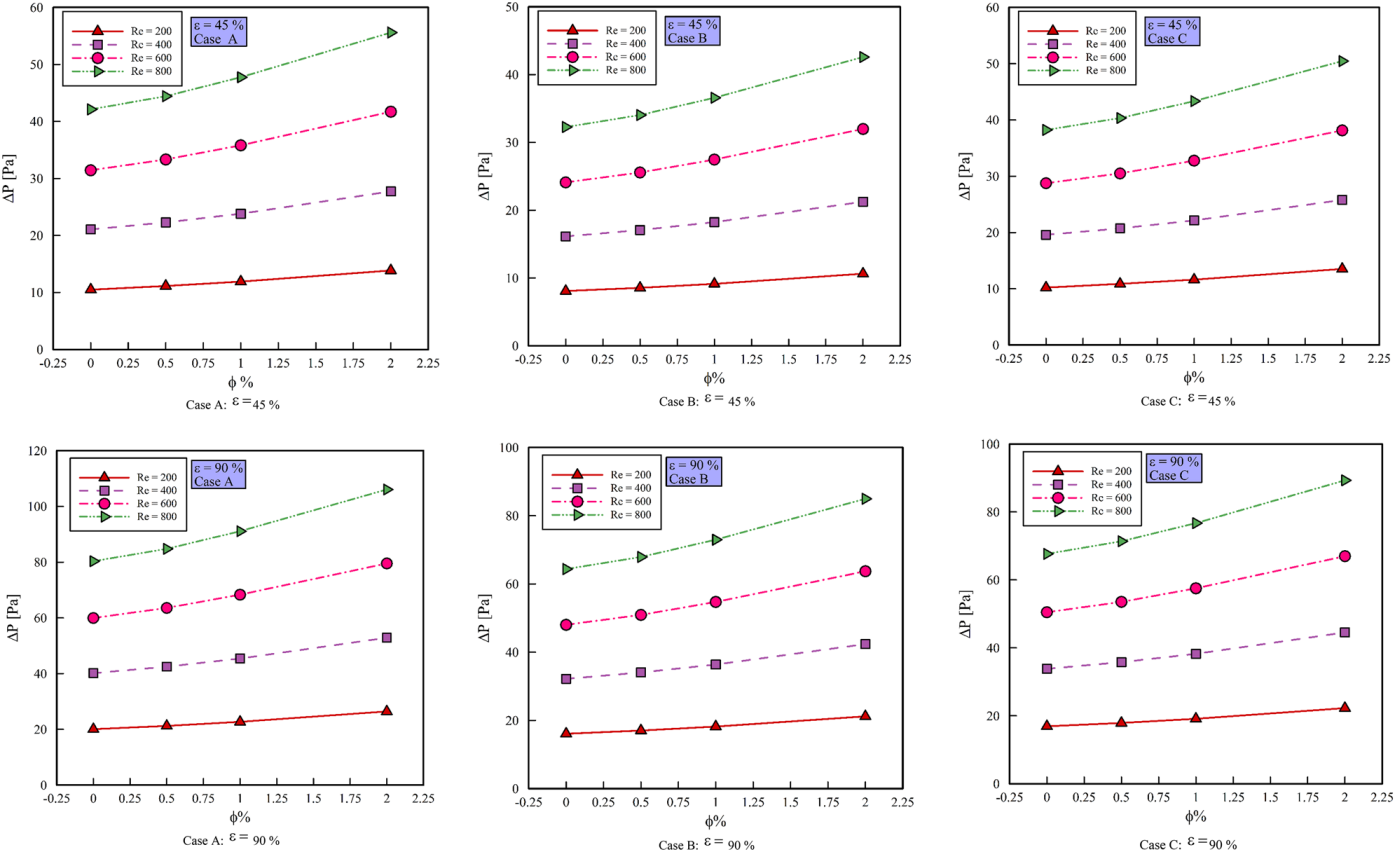
$$\Delta P$$ versus $$\phi$$.

Figure 7 shows the $\Delta P$ versus the $\phi$. Increasing the $\phi$ increases the density of the working fluid and due to the direct relationship between the Reynolds number and the density of the working fluid, with increasing the Reynolds number, the $\Delta P$ also increases. Comparing different geometric cases, it can be said that case C, which increases the Reynolds number locally by rotating the cylinders, ultimately increases the $\Delta P$ compared to case B. On the other hand, in case B, the presence of a step inside the two-dimensional microchannel increases the cross-sectional area and decreases the Reynolds number, which is a significant decrease, and in general, in case B, we see less $\Delta P$ than case A. By comparing the $\Delta P$ in different Reynolds numbers, we can say that due to the increase in the number of input Reynolds and consequently the inlet speed, we see more $\Delta P$ in all geometric cases. Comparing porosity percentages with each other, the $\Delta P$ at $\varepsilon$ = 90% is greater than the $\Delta P$ at $\varepsilon$ = 45%. The reason for this phenomenon is that there is more free space at $\varepsilon$ = 90% for the passage of nanofluids and the movement of nanofluids is less with obstacles and the velocity is higher and as a result, the $\Delta P$ in $\varepsilon$ = 90% is higher than $\varepsilon$ = 45%.

### Effect of $\phi$ on $C_{f,ave}$

**Figure 8:**
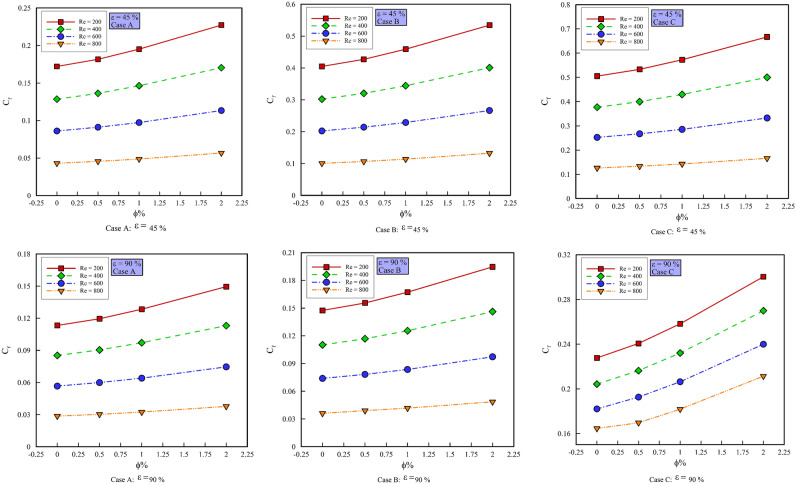
$$C_{f,ave}$$ versus $$\phi$$.

Figure 8 shows the $C_{f,ave}$ versus $\phi$, at different Reynolds numbers, porosity percentages, and geometric cases. As can be seen, with increasing the $\phi$, the $C_{f,ave}$ increases. This is due to the increase in the density and viscosity of the nanofluid, which increases the $C_{f,ave}$. Comparing different geometric cases, it becomes clear that the highest values of the $C_{f,ave}$ have the geometric case C. The reason for this phenomenon is the creation of velocity gradients near the wall due to the rotational motion of the cylinders. On the other hand, geometric case B shows a higher $C_{f,ave}$ than geometric case A due to the presence of steps and increase in velocity gradients. By comparing the porosity percentages with each other, it is concluded that due to the greater contact of the nanofluid with the porous medium and the creation of higher velocity gradients, the porosity percentage is 45% and the values ​​of $C_{f,ave}$ are 90% higher than the porosity percentage. In Fig. 8, with increasing the $\phi$, the $C_{f,ave}$ in the geometric case C in Re = 200 increases by 5.221, 6.95, and 11.103%, respectively, in Re = 400 increases by 5.962, 6.949 and 11.453, in Re = 600 increases by 5.499, 6.408 and 11.116% respectively and in Re = 800 increases by 5.354, 6.196 and 11.813% respectively. By increasing the $\phi$, the $C_{f,ave}$ in the geometric case B increases in Re = 200 by 5.193, 6.977 and 11.078%, respectively, in Re = 400 increases by 5.663, 6.892 and 11.157%, respectively, in Re = 600 increases by 531.5, 6.376 and 11.249% respectively and in Re = 800 increases by 7.548, 6.436 and 11.984% respectively. The $C_{f,ave}$ in the geometric case A with increasing the $\phi$ in Re = 200 increases by 5.69, 7.313 and 16.311%, respectively, in Re = 400 increases by 5.892, 7.292 and 16.296%, respectively, in Re = 600 increases by 5.819, 7.115 and 16.283% respectively and in Re = 800 it increases by 3.11, 7.155 and 16.261%, respectively.

### Effect of $\phi$ on $Nu_{ave}$

**Figure 9:**
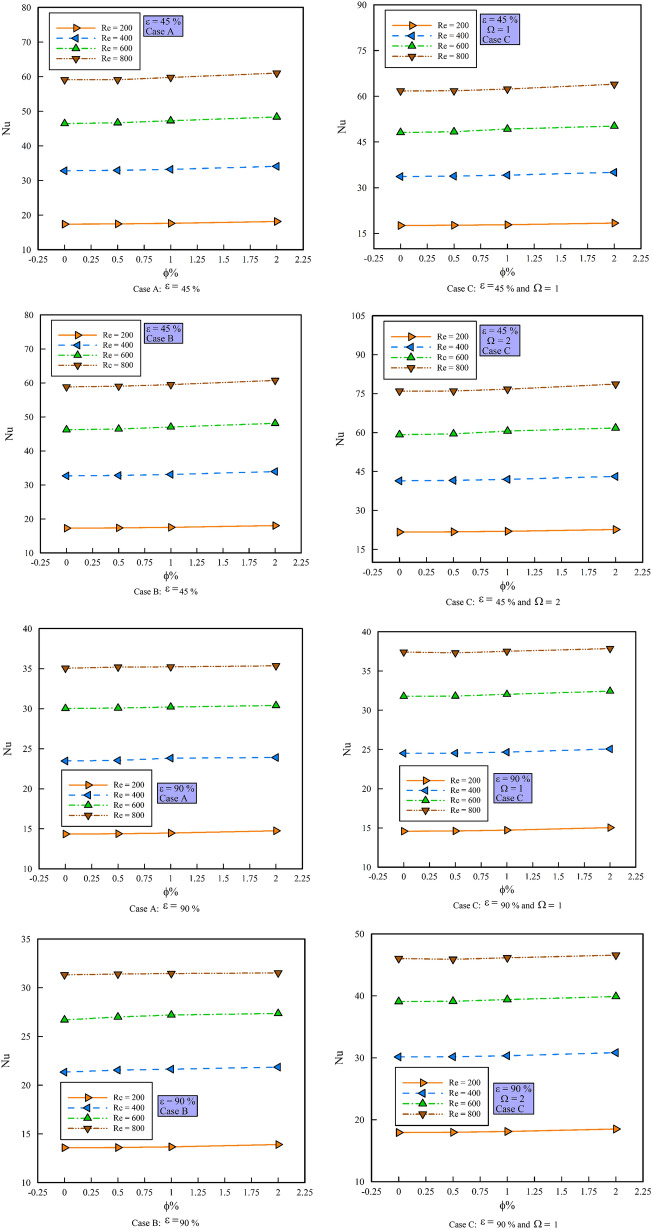
$$Nu_{ave}$$ versus $$\phi$$.

Figure 9 shows the $Nu_{ave}$ versus $\phi$ in different Reynolds numbers, porosity percentages, and geometric cases. As can be seen, with increasing $\phi$ and Reynolds number, the $Nu_{ave}$ increases. This behavior can be examined from both mathematical and physical perspectives. As the Reynolds number increases, the velocity increases, and thus the thickness of the thermal boundary layer decrease. From a mathematical point of view, reducing the temperature difference, which is associated with decreasing the thickness of the thermal boundary layer, increases the $Nu_{ave}$. In general, the $Nu_{ave}$ indicates the ratio of convection to conduction heat transfer. For this reason, in convection heat transfer, this ratio is always greater than one. Due to the increase in surface area, which itself increases the convective heat transfer coefficient, the $Nu_{ave}$ in $\varepsilon =$ 45% is higher than $\varepsilon =$ 90%. Comparing different geometries with each other, it can be seen that the geometric case B shows lower $Nu_{ave}$ values ​​than geometric case A due to the presence of steps and local decrease of Reynolds number. However, this is not the case for geometric case C with different non-dimensional rotational velocities because the rotational motion increases the Reynolds number locally and consequently the local velocity and shows higher values of the $Nu_{ave}$ than the geometric case A. Also, in geometric case C, with the increase of the non-dimensionless rotational velocity of the rotating cylinders, we see an increase of the local Reynolds number more than before, and this causes a further increase of the $Nu_{ave}$.

### Performance Evaluation Criterion (PEC)

**Figure 10:**
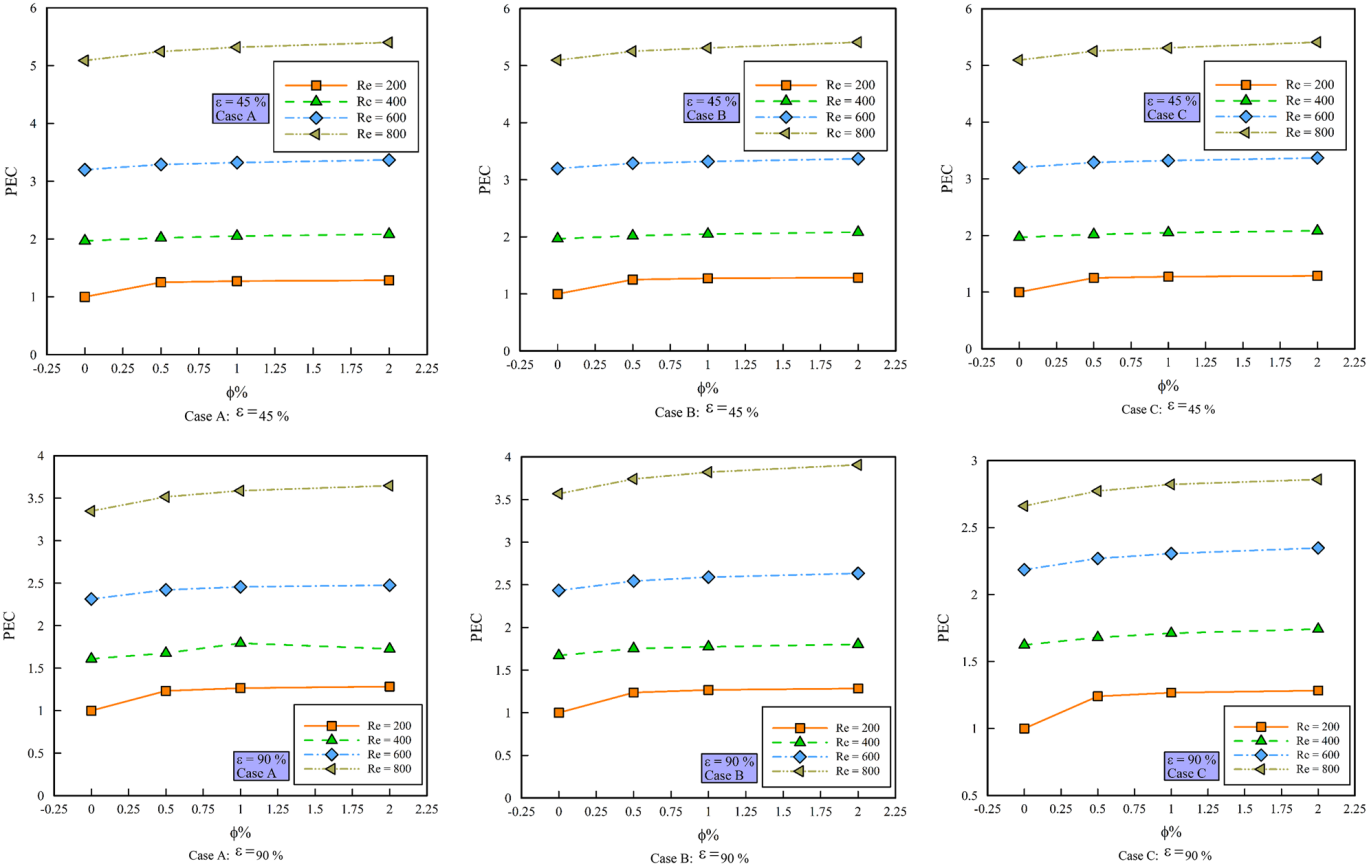
PEC versus $$\phi$$.

Figure 10 shows the PEC versus $\phi$ in different Reynolds numbers, porosity percentages and geometric cases. For the above reasons, increasing the porosity percentage reduces the heat transfer and consequently the PEC values. Placing the rotating cylinders in geometric case C causes the PEC to have an increasing trend compared to other geometric cases. In general, values ​​of PEC above 1 are known as favorite PEC values. As can be seen, all the values ​​in the mentioned figures have values higher than one, although the PEC changes with the $\phi$ are small. Increasing the $\phi$ in the geometric case A increases the PEC in Re = 200 by 20.12, 1.67 and 1.074%, respectively, in Re = 400 by 2.49, 1.518 and 1.563%, respectively, in Re = 600 is 2.758, 0.954 and 1.404%, respectively, and in Re = 800 is 2.982, 1.399 and 1.338%, respectively. Increasing the $\phi$ in geometric case B increases PEC in Re = 200 by 20.139, 1.657 and 1.065%, respectively, in Re = 400 by 2.83, 1.328 and 1.556%, respectively, in Re = 600 by 2.766, 1.054 and 1.397%, respectively, and in Re = 800 by 2.891, 1.072 and 1.867%, respectively.

### Effect of $\phi$ on entropy generation

**Figure 11:**
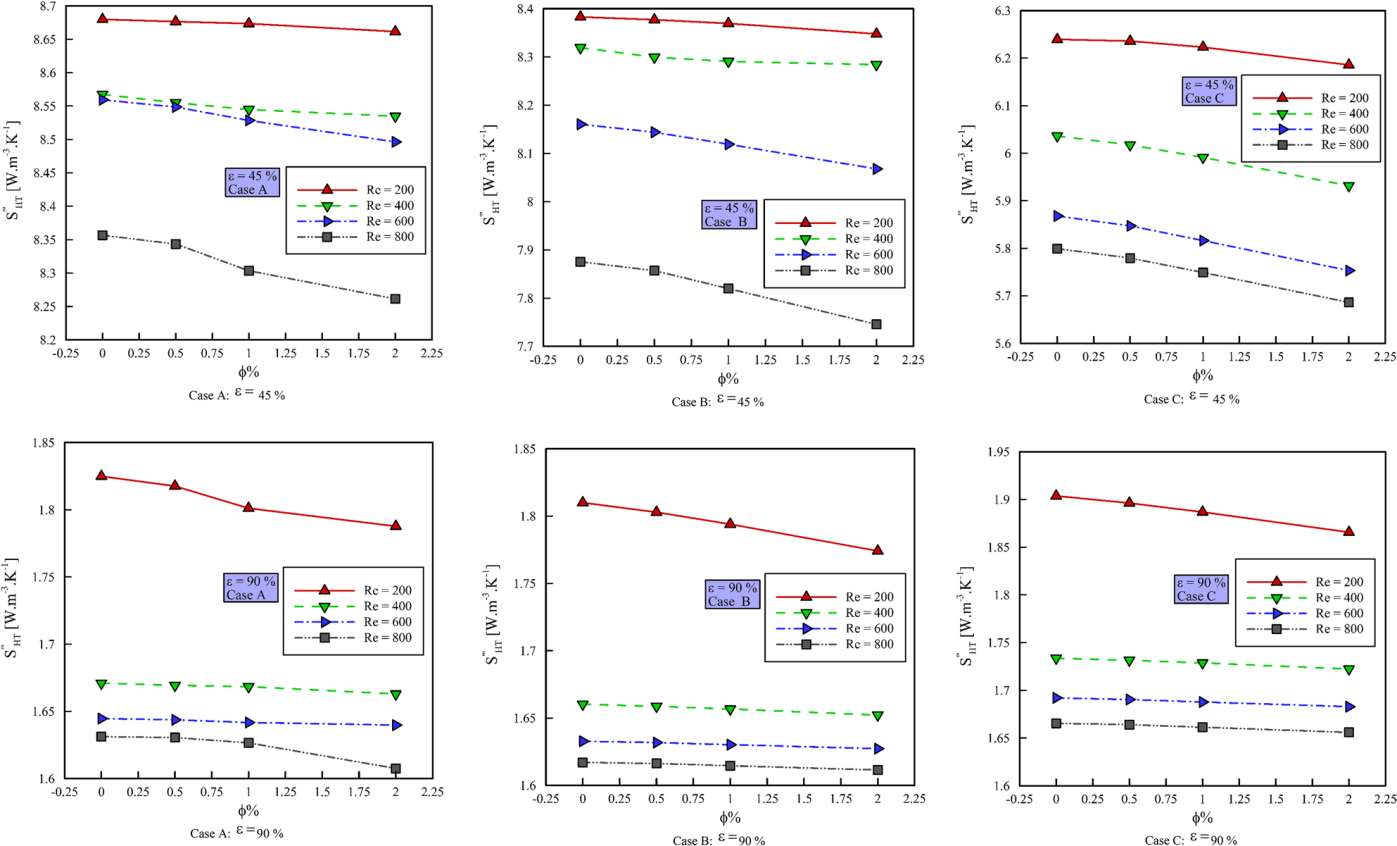
$S_{gen,HT}$ versus $\phi$ in different Reynolds numbers and porosity percentages.

**Figure 12:**
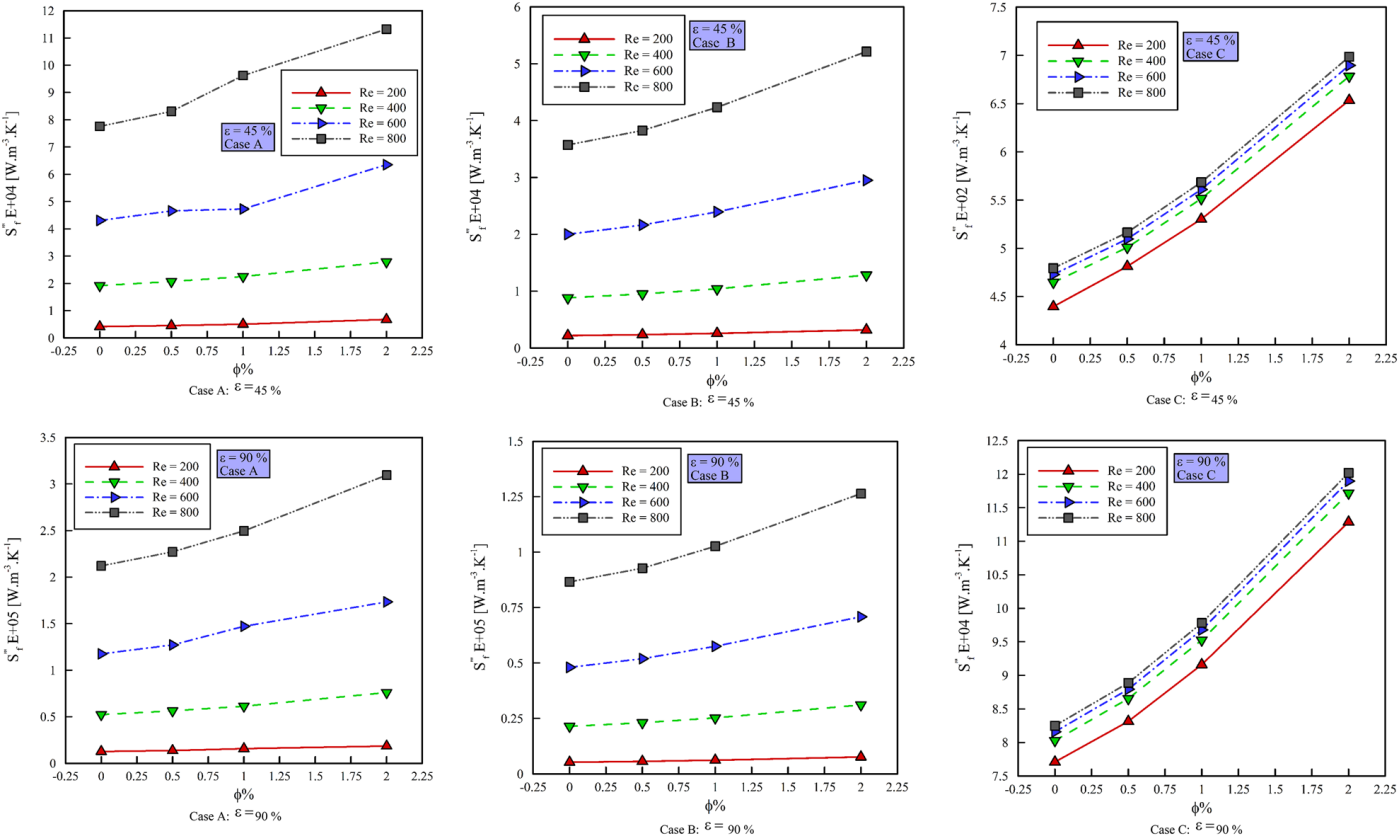
$S^{\prime\prime\prime}_{gen,f}$ versus $\phi$ in different Reynolds numbers and porosity percentages.

Figure 11 shows the entropy generation due to heat transfer ($S_{gen,HT}^{{{\prime \prime \prime }}}$) versus $\phi$ and Fig. 12 shows the entropy generation due to friction ($S_{gen,f}^{{{\prime \prime \prime }}}$) versus $\phi$. All the figures are presented at different Reynolds numbers, porosity percentages, and geometric cases. As can be seen, with increasing $\phi$ and Reynolds number, $S_{gen,HT}$ decreases. This is due to the reduction of the thermal boundary layer and the reduction of temperature gradients. As can be seen, increasing the Reynolds number and the $\phi$ increases the $S_{gen,f}^{{{\prime \prime \prime }}}$. In general, increasing the porosity percentage reduces $S_{gen,f}^{{{\prime \prime \prime }}}$ and $S_{gen,HT}$. Increasing the $\phi$ in case A reduces the $S_{gen,HT}$ in Re = 200 by 0.402, 0.906 and 0.741%, respectively, in Re = 400 by 0.945, 0.599 and 0.324%, respectively, in Re = 600 by 0.577, 0.121 and 0.116% respectively and in Re = 800 by 0.041, 0.244 and 1.169% respectively. Increasing the $\phi$ in geometric case B reduces the $S_{gen,HT}$ in Re = 200 by 0.391, 0.495 and 1.119%, respectively, in Re = by 095, 0.119 and o.272%, respectively, in Re = 600 by 0.064, 0.094 and 0.178%, respectively, and in Re = 800 by 0.05, 0.1 and 0.2%, respectively. Increasing the $\phi$ in the geometric case B increases the $S_{gen,f}^{{{\prime \prime \prime }}}$ in Re = 200 by 7.231, 9.461 and 23.39%, respectively, in Re = 400 by 7.19, 9.41 and 23.33%, respectively, in Re = 600 by 7.57, 10.678 and 23.171%, respectively, and in Re = 800 by 6.631, 10.667 and 23.147%, respectively. Increasing the $\phi$ in the geometric case C increases the $S_{gen,f}^{{{\prime \prime \prime }}}$ in Re = 200 by 8.654, 9.235 and 18.847%, respectively, in Re = 400 by 7.227, 9.162 and 18.696%, respectively, in Re = 600 by 7.215, 9.156 and 18.649% respectively and in Re = 800 by 7.185, 9.143 and 18.621%, respectively.

### Study of flow physics

**Figure 13:**
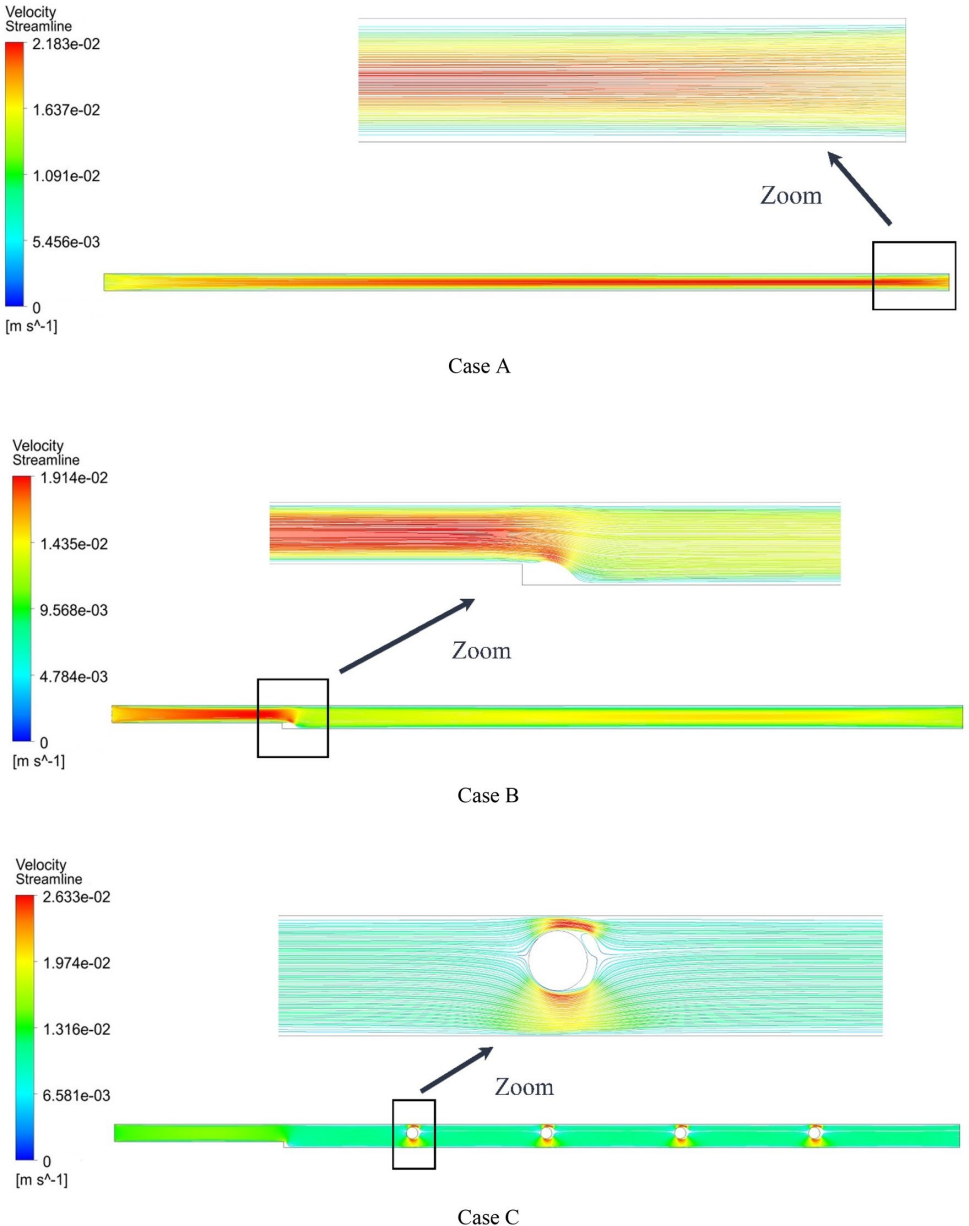
Streamlines in Re = 800, $\phi =$ 2.

**Figure 14:**
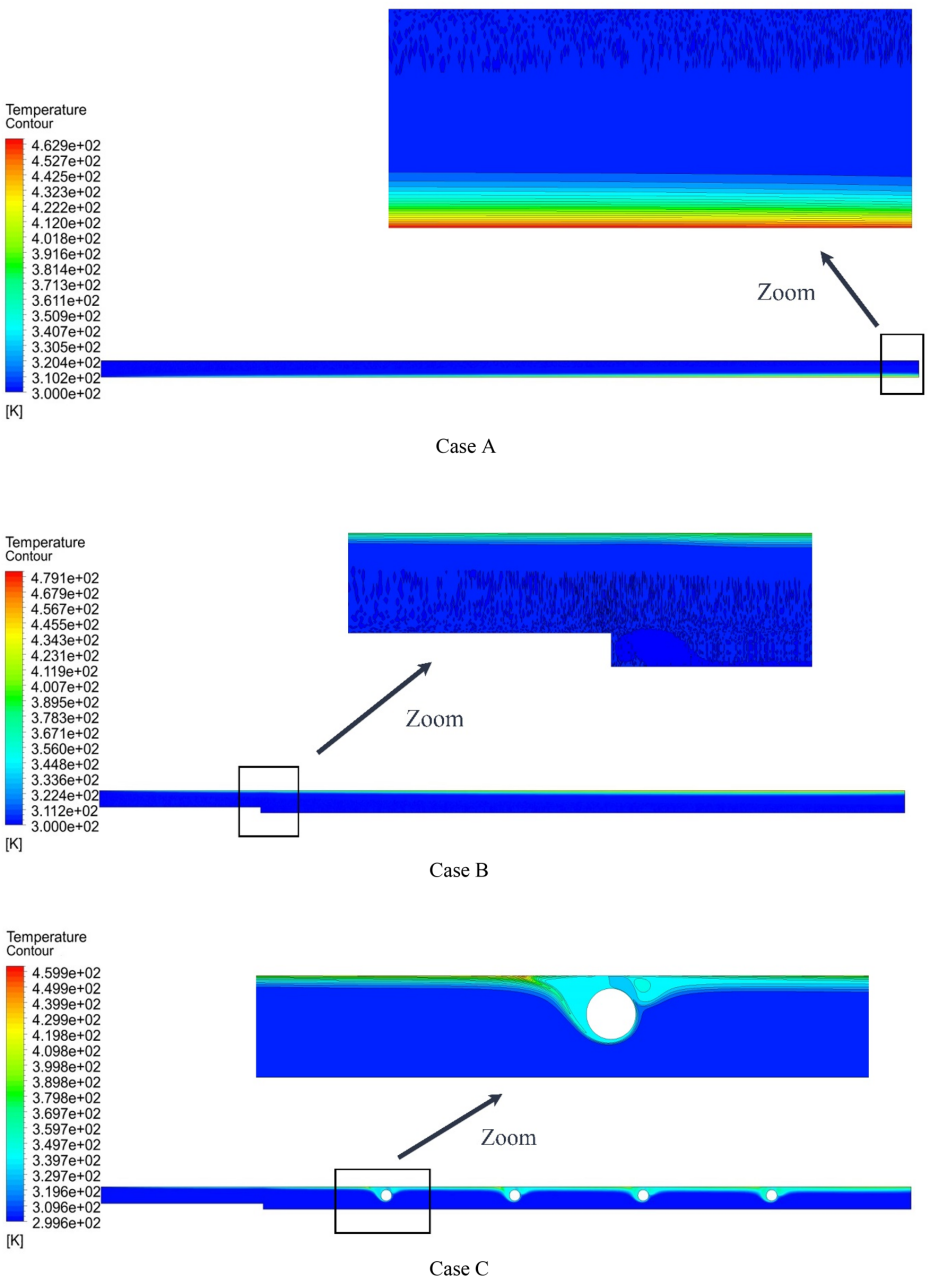
Temperature contours in Re = 800, $\phi =$ 2%.

**Figure 15:**
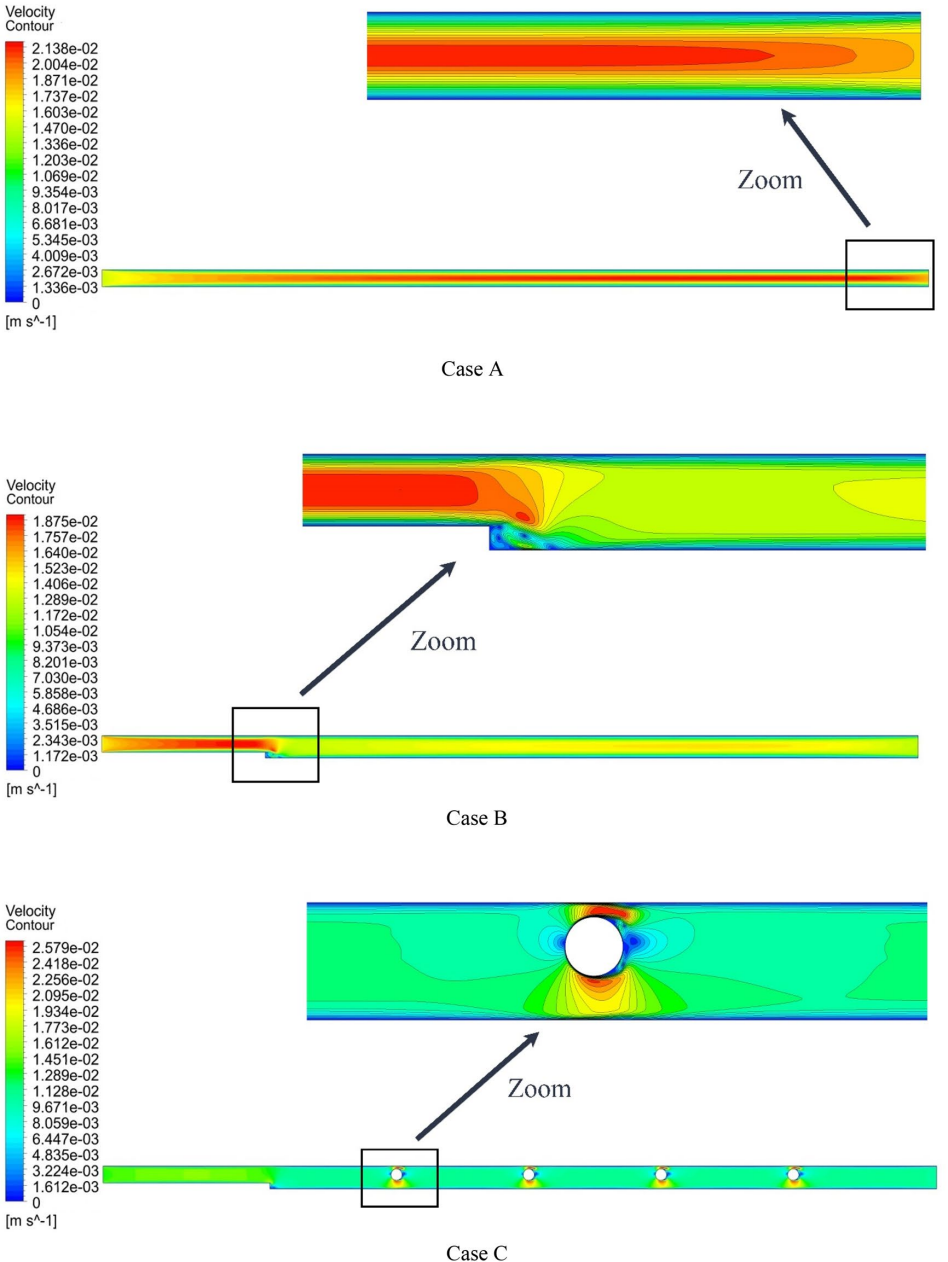
Velocity contours at Re = 800, $\phi =$ 2%.

Figure 13 shows the streamlines at Re = 800, $\phi$ = 2%, and various geometric cases. As can be seen, the streamlines in the middle of the microchannel have a different color from the streamlines adjacent to the microchannel wall. By adding steps and placing rotating cylinders, the shape of the streamlines changes. Figure 14 shows the temperature contour at Re = 800, $\phi$ = 2%, and various geometric cases. As can be seen, placing the rotating cylinders inside the microchannel disturbs the thermal dissipation pattern relative to the geometric case A. Figure 15 shows the velocity contour at Re = 800, $\phi$ = 2%, and various geometric cases. By placing the step in the microchannel, the flow pattern changes, and the local velocity decreases. Also, placing the rotating cylinders in the microchannel causes the amount of velocity around the cylinders to change.

## Conclusion

The results extracted from the numerical simulation include the following:

- Increasing the Reynolds number increases the heat transfer.
- The use of nanoparticles improves the thermophysical properties of the base fluid.
- Reducing the porosity percentage reduces the heat transfer because the contact surface decreases.
- Increasing the Reynolds number leads to a decrease in the $C_{f,ave}$.
- Increasing the $\phi$ increases the $C_{f,ave}$ due to the increase in viscous forces.
- Increasing the $\phi$ has little effect on $S_{gen,HT}^{{{\prime \prime \prime }}}$.
- Increasing the dimensionless rotational velocity of rotating cylinders increases the $Nu_{ave}$.
- Increasing the $\phi$ does not have much effect on $Nu_{ave}$.

## List of symbols

$$C_{f}$$: Friction factor
$$C_{P}$$: Specific heat $$[J\,kg^{ - 1} \,K^{ - 1} ]$$
$$D_{h}$$: Hydraulic diameter $$[m]$$
$h\left( x \right)$: Local heat transfer coefficient $[W\,m^{ - 2} \,K^{ - 1} ]$
$h$: Average heat transfer coefficient $[W\,m^{ - 2} \,K^{ - 1} ]$
$$k$$: Thermal conductivity $$[W\,m^{ - 1} \,K^{ - 1} ]$$
K: Permeability of porous medium $[m^{ - 1} ]$
L: Characteristic length $$[m]$$
$$\Delta p$$: Pressure drop $$[Pa]$$
$${\text{Re}}$$: Reynolds number
$S_{T}^{{\prime\prime\prime}}$: Entropy generation due to heat transfer $[W\,m^{ - 3} \,K^{ - 1} ]$
$S_{F}^{{\prime\prime\prime}}$: Entropy generation due to friction $[W\,m^{ - 3} \,K^{ - 1} ]$
$S^{{\prime\prime\prime}}$: Total entropy generation $[W\,m^{ - 3} \,K^{ - 1} ]$
$$T_{b}$$: Bulk temperature $$[K]$$
$$T_{w}$$: Wall temperature $$[K]$$
u,v: Velocity vector components $\left[ {m\,s^{ - 1} } \right]$
